# Bridging Data Models in Health Care With a Novel Intermediate Query Format for Feasibility Queries: Mixed Methods Study

**DOI:** 10.2196/58541

**Published:** 2024-10-14

**Authors:** Lorenz Rosenau, Julian Gruendner, Alexander Kiel, Thomas Köhler, Bastian Schaffer, Raphael W Majeed

**Affiliations:** 1 IT Center for Clinical Research, University of Lübeck, Gebäude 64, 2.OG, Raum 05, Ratzeburger Allee 160, Lübeck, 23562, Germany, 49 451 3101 5636; 2 Chair for Medical Informatics, Friedrich-Alexander-Universität Erlangen-Nürnberg, Erlangen, Germany; 3 Leipzig Research Centre for Civilization Diseases, University of Leipzig, Leipzig, Germany; 4 Federated Information Systems, German Cancer Research Center (DKFZ), Heidelberg, Germany; 5 Complex Medical Informatics, Medical Faculty Mannheim, Heidel­berg University, Mannheim, Germany; 6 Mannheim Institute for Intelligent Systems in Medicine, Medical Faculty Mannheim, Heidel­berg University, Mannheim, Germany; 7 Institute for Medical Informatics, University Clinic Rheinisch-Westfälische Technische Hochschule Aachen, Aachen, Germany

**Keywords:** feasibility, FHIR, CQL, eligibility criteria, clinical research, intermediate query format, healthcare interoperability, cohort definition, query, queries, interoperability, interoperable, informatics, portal, portals, implementation, develop, development, ontology, ontologies, JSON

## Abstract

**Background:**

To advance research with clinical data, it is essential to make access to the available data as fast and easy as possible for researchers, which is especially challenging for data from different source systems within and across institutions. Over the years, many research repositories and data standards have been created. One of these is the Fast Healthcare Interoperability Resources (FHIR) standard, used by the German Medical Informatics Initiative (MII) to harmonize and standardize data across university hospitals in Germany. One of the first steps to make these data available is to allow researchers to create feasibility queries to determine the data availability for a specific research question. Given the heterogeneity of different query languages to access different data across and even within standards such as FHIR (eg, CQL and FHIR Search), creating an intermediate query syntax for feasibility queries reduces the complexity of query translation and improves interoperability across different research repositories and query languages.

**Objective:**

This study describes the creation and implementation of an intermediate query syntax for feasibility queries and how it integrates into the federated German health research portal (Forschungsdatenportal Gesundheit) and the MII.

**Methods:**

We analyzed the requirements for feasibility queries and the feasibility tools that are currently available in research repositories. Based on this analysis, we developed an intermediate query syntax that can be easily translated into different research repository–specific query languages.

**Results:**

The resulting Clinical Cohort Definition Language (CCDL) for feasibility queries combines inclusion criteria in a conjunctive normal form and exclusion criteria in a disjunctive normal form, allowing for additional filters like time or numerical restrictions. The inclusion and exclusion results are combined via an expression to specify feasibility queries. We defined a JSON schema for the CCDL, generated an ontology, and demonstrated the use and translatability of the CCDL across multiple studies and real-world use cases.

**Conclusions:**

We developed and evaluated a structured query syntax for feasibility queries and demonstrated its use in a real-world example as part of a research platform across 39 German university hospitals.

## Introduction

### Background

In the rapidly evolving field of medical research, patient data have emerged as a critical resource. The vast amounts of data generated through clinical encounters, laboratory tests, imaging studies, and other patient interactions hold the potential to significantly advance our understanding of disease processes and treatment outcomes. Clinical Data Repositories (CDRs) are a valuable tool for storing, organizing, and retrieving this wealth of patient data. These repositories facilitate data storage in a structured and standardized manner, enabling researchers to query these data efficiently for various research purposes.

One key aspect of effectively using CDRs is the ability to perform feasibility queries. These queries allow researchers to assess the availability and adequacy of data for specific research questions before embarking on full-scale studies. Doing so can save considerable time and resources by identifying potential issues, such as insufficient sample size or a lack of necessary data elements.

### Distributed Data Collections

The landscape of data repositories is not homogeneous. There are 2 primary approaches to data repository management: the classical single repository approach and the federated approach. Traditionally, these repositories have been centralized, pooling data from various sources into a single repository [1]. However, this classical approach has been challenged by the emergence of federated data repositories [1,2].

The classic single repository approach involves a centralized system where all data are stored and managed in one place. This solution offers the advantage of uniformity and ease of data management. It enables efficient data quality benchmarking at scale and the generation of derivatives, harmonized variables, and units of measure for comparable and consistent analytics [1]. However, it is often impractical or impossible to implement, especially when dealing with multiple institutions, each having its own schema for its clinical data repository.

On the other hand, the federated approach involves a network of repositories, each maintained by different institutions. These repositories operate independently but are interconnected for data sharing and collaboration. The data generally remain at the generating site, which offers the advantages of local curation by personnel deeply familiar with the data [1] and maintains data anonymity and security [2]. The data can then be analyzed using a federated approach or, if the correct patient consent is given, be transferred to a central data management unit for a specific analysis.

This approach respects individual institutions’ autonomy and data governance policies, making it a more feasible option for multi-institutional collaborations [3-9] and can enhance the scope and depth of clinical research by enabling access to a broader range of data.

Despite the potential benefits of federated data repositories, performing feasibility queries across multiple CDRs presents significant challenges [10]. Each repository contains data originating from different source systems, leading to heterogeneity in data formats, terminologies, and quality. This heterogeneity can significantly complicate the process of data integration and harmonization, making it challenging to perform comprehensive and accurate feasibility queries [10].

Moreover, the federated nature of the system introduces additional complexities. Data privacy regulations and institutional policies may restrict the sharing and use of certain data, further complicating the query process. Additionally, the technical infrastructure required to support secure and efficient data exchange across multiple repositories can be challenging to implement and maintain.

### Data Exchange Standards for Interoperability

In a federated network, the commitment to an interoperability standard becomes pivotal to tackling these challenges. Prominent examples include but are not limited to Fast Healthcare Interoperability Resources (FHIR) [11], OMOP CDM [12], i2b2 [13], and OpenEHR [14] share the commonality of being centered around the patients’ medical history.

Agreeing on an interoperability standard only partially solves the challenge. While a health care data exchange standard facilitates the conversion of existing data into a common format at each hospital, a distributed feasibility query platform for the data is still missing.

### Tools for Feasibility Queries

Besides the data integration standardization, interactive user interfaces enable researchers to design and submit feasibility queries. For this purpose, a multitude of tools for feasibility queries exist (eg, i2b2 [13,15], TriNetX [16], tranSMART [17], SampleLocator [18-20], Observational Health Data Science and Informatics [OHDSI] ATLAS [21], DZHK Feasibility Explorer [22]), each with its own data formats, standards, and query languages, including Structured Query Language (SQL), Clinical Quality Language (CQL), FHIR-Search, and Archetype Query Language (AQL). Consequently, querying across these different tools is difficult as there is no common query representation, and researchers must navigate these diverse tools and formats, particularly when dealing with cross-institutional data or distributed data storage within an institution.

Within the broader context of establishing a feasibility platform as part of the central German Portal for Health Data (FDPG), this research introduces a novel query syntax, serving as an intermediary between user interfaces and data repositories. This syntax is designed to be sufficiently flexible to ensure interoperability while maintaining simplicity. It focuses on the primary needs of a feasibility query, while allowing the syntax to be translated into repository-specific languages like FHIR-Search or CQL.

Our approach is grounded in the broader context of clinical research, where the reuse of eligibility criteria is common, whether in their original form or with modifications. These criteria are instrumental not just for feasibility studies but also for prescreening, data selection, extraction, and validation. Consequently, a need has emerged to decouple the representation of eligibility criteria from their implementation in specific systems. A mechanism to express complex criteria and combinations thereof in a way that is both intuitive and adaptable to varying implementation needs is required.

In this study, we describe the development and application of the query syntax within the network of the Medical Informatics Initiative (MII), encompassing 39 German university hospitals, specifically, the FDPG feasibility platform and show how it achieves interoperability across different research platforms.

## Methods

### Requirement Analysis

In our pursuit to create an intermediate query syntax to express eligibility criteria, we performed a requirement analysis. Within it, we combine insight from feasibility queries and cohort selection, with the latter often manifesting as a query output in the form of cohort size rather than a list of discrete patient identifiers.

Our research reviewed existing tooling, namely i2b2, TriNetX, and OHDSI Atlas. We aimed to identify common functionalities and essential features across these tools. To obtain insight into the criteria’s structure and complexity, we analyzed existing eligibility criteria from ClinicalTrials.gov [23] and incorporated the findings from Ross et al [24] and Gulden et al [25]. Moreover, we integrated insights from the usability study by Schüttler et al evaluating feasibility tools [26], conducted expert interviews and recursively synchronized the requirements within our project. This multifaceted analysis allowed us to infer a set of requirements crucial for developing our query syntax. These requirements were categorized into query expressiveness, interoperability, and accessibility.

#### Expressiveness Requirements

The query syntax should:

1. allow for the definition of inclusion and exclusion criteria
2. be expressed in Boolean logic.
3. allow the expression of exclusion criteria.
4. support at least patient as query subject (feasibility queries can be performed on different query subjects: find all patients with specific criteria, find all encounters with specific criteria, find all specimens with specific criteria).
5. use unique identifiers for criteria and concepts.
6. support the following filter on the criterion level:
  - existence of a criterion
  - numeric restriction
  - concept filter
  - time restrictions
  - attribute filters

#### Interoperability and Accessibility Requirements

The query syntax should:

1. provide an abstract (decoupling) layer between the user interface and the query execution.
2. have a low level of complexity and be easily translatable to different query languages.
3. be suitable for integration with the Health Level Seven International (HL7) FHIR standard used by the MII.
4. use a widely used data exchange format like JSON to ease parsing and generation
5. human readability or writability
6. ideally directly support the use of standard medical terminology (LOINC [Logical Observation Identifiers Names and Codes], SNOMED-CT [Systematized Nomenclature of Medicine–Clinical Terms], *ICD-10* [*International Statistical Classification of Diseases, Tenth Revision*], etc) to lower mapping efforts

### Related Work and Existing Solutions

Analyzing the existing solutions, we found that none of the solutions met all the requirements. Most failed to have a formally defined low-complexity feasibility query syntax, and i2b2 was missing the direct relationship with the terminology on the syntax level. FHIR Search and the FHIR standard did not provide the ability to express a feasibility query in the required scope [25] at the time of our research. Other query languages that could have been candidates, like CQL or SQL, are complex or data model specific, making the translation between different data models and their representation, as well as the generation of the syntax by a user interface, challenging.

### Evaluation

To evaluate the specification of the query syntax, we compared the final specification with our requirements and additionally demonstrated its applicability beyond the scope of FHIR by applying it to AQL.

We incorporated the solution into a large-scale real-world distributed feasibility query infrastructure, including a user interface, where it was integrated as the central intermediate query syntax. We further evaluated the applicability of the syntax to a wide range of clinical criteria and investigated its translatability, as well as how well it lends itself to creating a user interface for feasibility queries. Beyond the use in our projects based on German data sets and specifications, we also successfully applied the Clinical Cohort Definition Language (CCDL) to the international Synthea [27] data set.

### Ethical Considerations

No ethics board decision is required as we are presenting a technical solution without working on patients’ data.

## Results

Based on the requirements of a team of experts, we created the “Clinical Cohort Definition Language,” an intermediate query syntax for feasibility queries. The exchange format for the syntax was chosen to be JSON, which is currently widely used across the software community and is familiar to software developers from user interfaces, REST application programming interfaces (APIs), and query execution backends alike.

### Criterion Types and Filters

The atomic component of CCDL is the criterion, serving as the foundational building block for inclusion or exclusion criteria. Each criterion is uniquely identified using a tuple of code system and code (which we named termCode) analogous to FHIR and OMOP-CDM (For conceptual equivalence between concepts across medical terminologies, multiple termCodes can be provided, eg, the criterion for sleep apnea may be represented by the termCodes G47.3 from *ICD-10* and 73430006 from SNOMED-CT). Each termCode may have an additional “display” attribute, which serves purely as a visual representation to make the interpretation of a CCDL easier for humans. Within our CCDL, the criteria can occur as 1 of 4 different base types of criteria:

- Exist criteria with no additional filters (eg, conditions or a laboratory concept with no filter, like the existence of a hemoglobin value regardless of the value)
- Comparatively restricted numerical criteria (eg, hemoglobin laboratory value <12 g/dL)
- Range-restricted numerical criteria (eg, hemoglobin laboratory value between 10 and 12 g/dL)
- Value set restricted criteria (eg, gender=female or male)

Additionally, each criterion can be further restricted to a date range (eg, a Condition that occurred between January 1, 2024, and February 5, 2024), and it supports additional “attribute” filters, which can be added to each of the base types of criteria. The attribute filters support similar filters that identify the criterion types, ie, comparative numerical, comparative range, and value set restriction (eg, the body site=skin for a tissue specimen—see Multimedia Appendix 1).

### The Explicit Logic Layer

**Figure 1.**
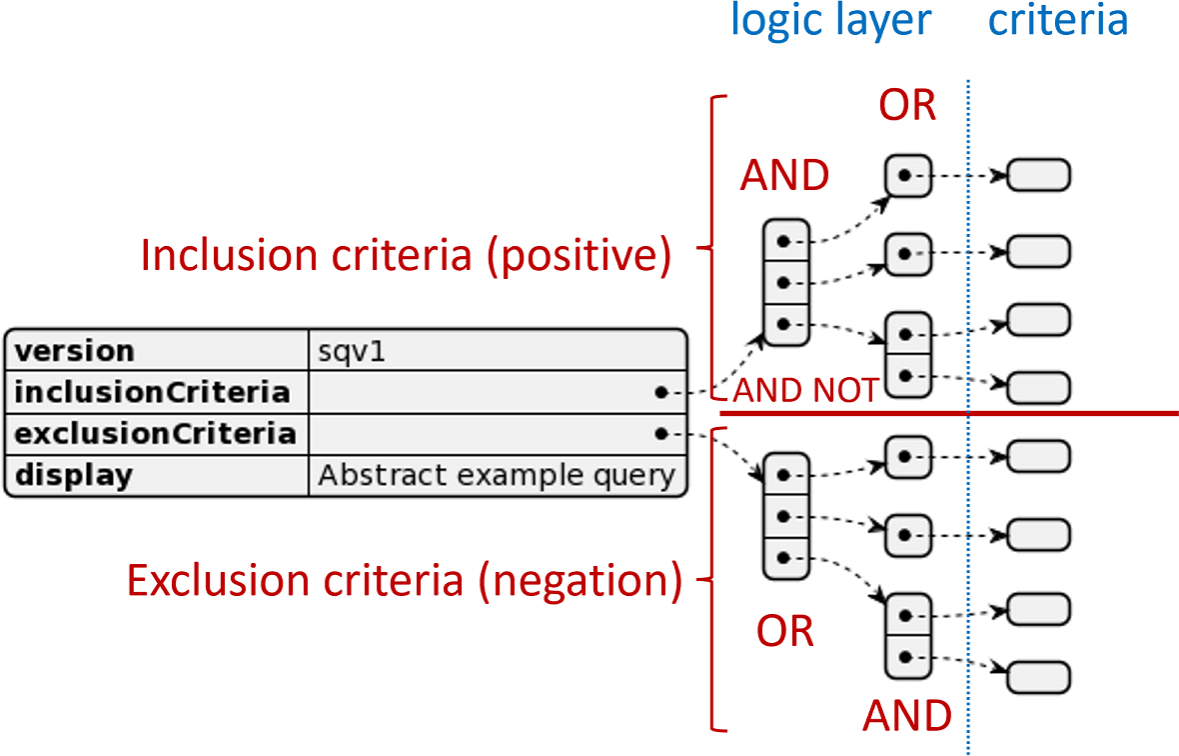
Structured query syntax top-level elements and logic layer. Certain criterion types will imply additional intrinsic logical relations. See ValueSet criteria and attribute filters and time restrictions.

The logic layer of the query aligns with existing solutions (i2b2/tranSMART/TriNetX) in representing the structured query as a combination of conjunctive normal form (CNF) and disjunctive normal form (DNF). Every criterion is embedded into the logic layer in a CNF for inclusion criteria and DNF for exclusion criteria (Figure 1). Inclusion and exclusion criteria are then logically combined via an AND NOT operator by subtracting the result of the exclusion criteria from the result of the inclusion criteria. Every feasibility query also receives a syntax version number and an additional description. The syntax version allows to distinguish the current version from future versions and changes, and the description allows the query to transport additional human-readable information about the query.

### The Implicit Logic (Criteria Expansion)

Apart from the explicit logic layer across criteria, different types of criteria and their filters further impact the execution logic as follows.

**Figure 2.**
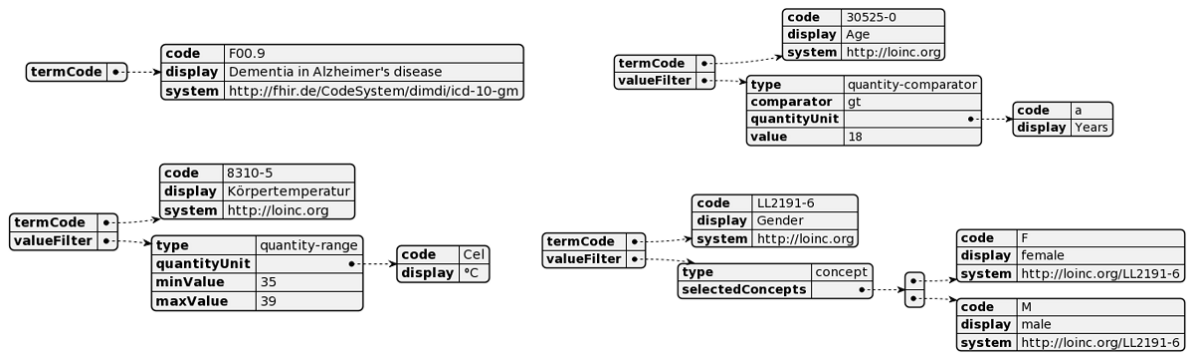
Different types of criteria definitions. (A) Simple conceptual criterion. (B) Numeric criterion with quantitative comparison. (C) Numeric criterion with range restriction. (D) ValueSet criterion.

ValueSet criteria (see Figure 2D) allow the selection of multiple values (concepts). In this case, the value selections are treated as OR choices. For example, gender = (male, female) expands to: (gender=male) OR (gender=female).

Attribute filters for each criterion are additional filters that can be set for each criterion. All individual filters on a criterion are combined using AND. For example, a specimen of type “Tissue specimen” and body site “skin” only applies to specimens with the type of Tissue and the body site skin.

The same applies to time restrictions. In this example, the time restriction “between 2020-01-01 and 2021-01-01” will predictably be added using an AND conjunction of the type, body site, and time restriction.

Furthermore, there is an implicit OR expansion of criteria when the criterion-identifying code is a parent code of multiple child codes within a terminology hierarchy. For example, suppose a researcher adds the diagnosis of type 2 diabetes mellitus as a criterion (*ICD-10* code=E11). In that case, it can be expanded to search all subtypes of type 2 diabetes mellitus (E11, E11.3, E11.31, E11.30, E11.1, E11.11, E11.0, E11.01, E11.7, E11.75, E11.74, E11.73, E11.72, E11.4, E11.41, E11.40, E11.8, E11.81, E11.80, E11.2, E11.21, E11.20, E11.5, E11.51, E11.50, E11.6, E11.61, E11.60, E11.9, E11.91, E11.90) combining them using a logical OR operation).

### Context-Dependent Criteria

In some cases, a criterion cannot be uniquely defined by its term code within a terminology, making it impossible to map a criterion for execution. One example of this is the use of *ICD-10* condition codes for causes of death, specimen-specific conditions, or the general condition of a patient.

In modern terminologies like SNOMED-CT, this can be resolved using postcoordination, where a combined code, which carries the context, is created. For example, 419620001|Death|:42752001|Due to|=22298006|Myocardial infarction| which, while in line with SNOMED Compositional Grammar [28], a template to express this is not currently part of the SNOMED-CT implementation.

**Figure 3.**
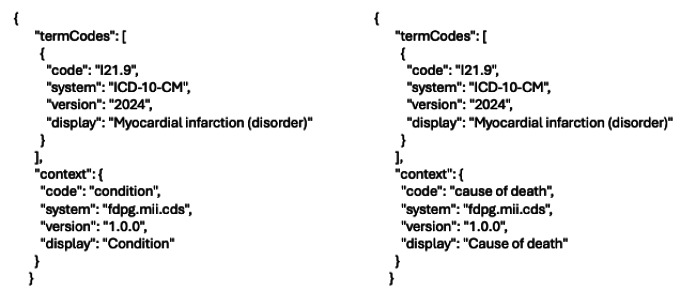
Myocardial infarction in 2 contexts (condition and cause of death).

The syntax we developed here allows for post-coordinated codes; however, we allow for an additional “context” attribute for some use cases where postcoordination is unsuitable. The context attribute is modeled after our termCode attribute and provides an extra term code to identify the context. Figure 3 provides an example for myocardial infarction as condition aand cause of death.

### Data Availability

As a technical solution to define the structure of the CCDL, we decided on the JSON Schema definition and made it publicly available [29]. The schema serves implementation guidance and validation purposes; the git repository also contains documentation examples, test data, and the capabilities to create matching test queries.

### Requirement Verification

An analysis was performed based on the structure defined in the JSON schema to evaluate the developed intermediate query syntax. The following table (Table 1) presents the detailed results of this analysis:

This syntax efficiently meets a wide range of expressiveness and interoperability requirements, demonstrating capabilities in defining complex medical queries with standard terminologies and logical operators.

**Table 1.** CCDL^a^ components and their purpose regarding the expressiveness requirements.

Component	Key properties	Purpose and function	Requirements met
inclusionCriteria	CNF^b^ without negation	Conjunction of criteria with logical operators.	Expressive query formulation, boolean logic
exclusionCriteria	DNF^c^ without negation	Allows negation of criteria for comprehensive exclusion.	Negation of criteria on a group level, Boolean logic
termCode	code, system, version, display	Identifies concepts using standard coding systems.	Standard medical terminology, uniqueness
criterion	context, termCodes, valueFilter, attibuteFilter, timeRestriction	Sets criteria with defined context, using term codes and filters.	Expressiveness of simple and complex eligibility criteria
timeRestriction	afterDate, beforeDate	Specifies time frame for criteria fulfillment.	Time restrictions
unit	code, display	Standardized unit definition, adhering to UCUM^d^ units.	Use of standardized units
valueFilter	type (concept, quantity-comparator, etc)	Varied filtering types for flexible data querying.	Numeric restriction, concept restrictions
attributeFilters	type (concept, quantity-comparator, reference)	Mechanism for detailed filtering at the attribute level.	Detailed filtering, clinical relations

a CCDL: Clinical Cohort Definition Language.

b CNF: conjunctive normal form.

c DNF: disjunctive normal form.

d UCUM: ___.

### Evaluation and Use of the CCDL in Real-World Scenarios

We believe the potential of the CCDL extends beyond its application in the federated feasibility portal of the German Research Portal for Health. Nevertheless, the CCDL remains a crucial technical solution within the FDPG’s feasibility portal.

**Figure 4.**
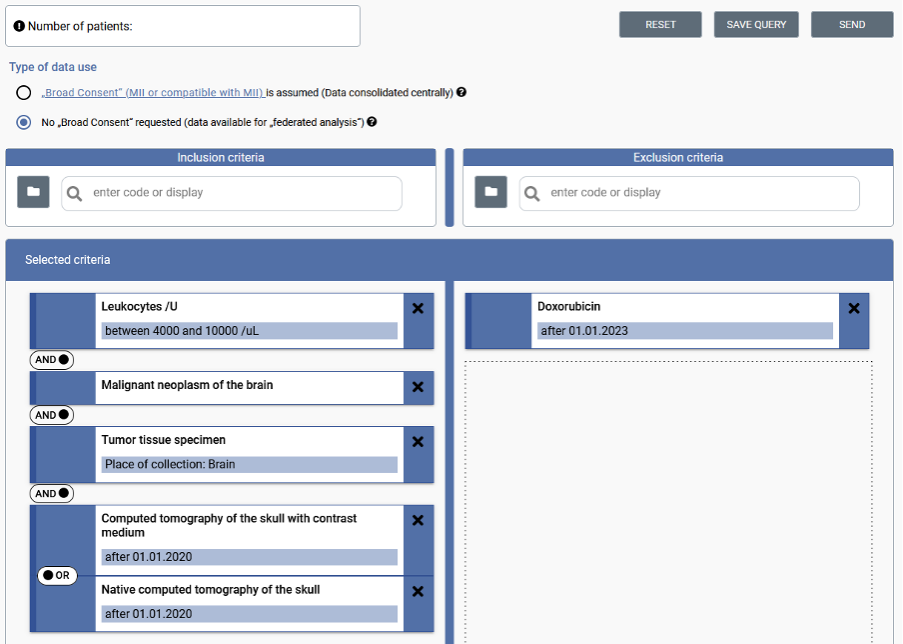
Example of a feasibility query in the central German Portal for Health Data (FDPG) feasibility portal to find patients with a leukocyte count within a normal range, with a malignant neoplasm of the brain, available tumor tissue specimen, and a CT scan after January 1, 2020, who did not take doxorubicin.

We created a user interface for the feasibility queries in the FDPG (Figure 4), which generates the CCDL, demonstrating how it lends itself well to building feasibility query user interfaces [30,31]. The CCDL especially supports this as its design follows the typical way of feasibility query creation as seen in platforms such as the FDPG, i2b2, OMOP, and TrinetX. We evaluated the usability of the user interface across multiple projects [32,33] and embedded it in a German-wide distributed research infrastructure [10,34]. These evaluations highlighted the applicability of the CCDL to a feasibility query and that the usability issues found were not due to a lack of expressiveness of the CCDL. We further used the Synthea data set to test the CCDL against [35], demonstrated the ability of the CCDL to represent a wide range of criteria [36], and showed that it could be fully translated to FHIR Search [37], CQL [38], and AQL [39]. At the time of writing, almost 9000 CCDLs have been created and executed across Germany.

## Discussion

### Principal Findings

We presented an intermediate feasibility query syntax that separates concerns between the user interface and the execution of a feasibility query on different research repositories and their specific query languages. The syntax defined here fulfills all the interoperability and accessibility requirements while supporting a broad range of expressiveness requirements we identified by analyzing existing query tools. The solution is fully compatible with established medical terminology standards, notation of parameters, and restriction semantics.

The solution we describe here is compatible with the query logic established by i2b2 and, therefore, tranSMART and TriNetX. This means that tools like i2b2 or similar could be easily extended to produce our syntax.

The CCDL was further used as part of a larger infrastructure for feasibility queries in Germany and is currently used as the interface for feasibility queries within the German research portal for health, supporting feasibility queries across 39 university hospitals in Germany. We successfully created translation components for FHIR Search and CQL in the current implementation. Current research also indicates the adaptability for FHIR Pathling’s aggregation API [40], and SQL. The criteria content and the required reintroduction of data model—dependent information are obtained from an automatically generated search ontology [36].

### Related Work

While the expression of eligibility criteria within a specific data model context is well established and adequately discussed in this work, research on a data-agnostic intermediate format for computable eligibility has been sparse in recent years.

Alper et al [41] closely align with our approach of representing eligibility criteria in a structured format, namely the FHIR EvidenceVariable, which currently does not directly support the representation of eligibility criteria but may be refined to do so. Presumably, an FHIR representation would provide a structure beyond the realm of the MII, which could add significant value and improve syntax interoperability. However, in the early stages, the challenges of adopting new solutions could have impeded the development presented here. Our ongoing communication with the HL7 working group, which focuses on Research Studies, gives us confidence that once a suitable FHIR Resource is established or adapted to meet the needs outlined in our publication, the established technical components could be efficiently modified to align with these changes. Parallels can be drawn to implementing structured eligibility criteria, as presented by Yuan et al [42] and Fang et al [43]. Their publications present a half-automated approach to generate feasibility queries based on free text study protocols from ClinicalTrials.gov [23]. Their system is built around the OHDSI data model and uses the concept IDs. After converting the free text criteria, they allow users to edit and download an intermediate representation in JSON format. Unfortunately, no clear implementation guidelines on the format are given by Yuan et al [42] and Fang et al [43]. However, recurring themes include differentiating inclusion and exclusion criteria and defining temporal constraints. To our knowledge, contrary to our approach, they do not allow for further restrictions beyond the value constraint on specific criteria.

### Limitations

The separation of concerns, which the CCDL provides, also leads to the need for a mapping to identify the correct way of translating the CCDL information model to the local information model and terminology. The mapping allows the link between the specific data model and the criterion as identified in the CCDL to be created. One example of this is that for FHIR Search, the mapping for a condition criterion identified by a specific *ICD-10* code C50.0 would provide the information that the condition is found in the end point “/Condition” and the search parameter for the term code is “code” – Leading to the translated FHIR Search URL:

[fhir-base-url]/Condition?code=http://fhir.de/CodeSystem/bfarm/icd-10-gm|C50.0.”

Further, additional information about the terminology is necessary to allow the selection of criteria within a terminology hierarchy, where the criterion resolves to multiple child criteria. Finally, this then requires the query executor and the CCDL-generating user interface to agree on criteria or terminology entries.

One common requirement currently not supported by the CCDL is temporal interdependencies between different criteria. Therefore, queries like a specific laboratory value within a certain period of diagnosis cannot be currently expressed using the CCDL.

We deliberately decided to delay the implementation of this extension as time dependencies significantly increase the complexity and performance requirements of any query execution.

The data model agnostic nature of the CCDL is inherently valuable. Its full potential—the capability to be used across different health care data models—requires more than technical translation. For cross-model query capability, the existence of the concepts in all target data models must be ensured.

### Future Work

The CCDL described here provides a good base to make feasibility queries possible across various research repositories and close the gap between the different research repositories and their access. We have demonstrated the applicability of the CCDL to FHIR Search, CQL, and AQL; however, more repositories and other query languages, such as SQL on FHIR, OHDSI OMOP, or i2b2 might be added in the future. Further, one could imagine how separating the query syntax and execution would theoretically allow one to query different internationally distributed repositories such as FHIR, OMOP-CDM, and i2b2 simultaneously. Additionally, the CCDL is currently limited in how much it can express, and new capabilities will be added in the future. In this pursuit of making the CCDL more expressive, any extension must be weighed against the added complexity and overhead it introduces.

### Conclusion

We presented a query syntax for medical feasibility queries, which creates an abstract layer between the user interface and the execution query language. We showed how it is flexible enough to be translated into different query languages and can be used to express various complex feasibility queries. The applicability of the query syntax was further demonstrated by embedding it into a large research project where it is used to query multiple millions of patients across 39 German university hospitals. The CCDL for feasibility queries will be extended in the future to allow more features, and we are currently working on a modified version for data selection and extraction.

## Supplementary material

10.2196/58541 Multimedia Appendix 1 Example of specimen criteria with an ICD-o-3 (International Classification of Diseases for Oncology, 3rd Edition) attribute indicating the location the specimen was taken from.

## Abbreviations

API: application programming interface
AQL: Archetype Query Language
CCDL: Clinical Cohort Definition Language
CDR: Clinical Data Repository
CNF: conjunctive normal form
CQL: Clinical Quality Language
DNF: disjunctive normal form
FDPG: central German Portal for Health Data
FHIR: Fast Healthcare Interoperability Resources
HL7: Health Level Seven International
*ICD-10*: *International Statistical Classification of Diseases, Tenth Revision*
LOINC: Logical Observation Identifiers Names and Codes
MII: Medical Informatics Initiative
SNOMED-CT: Systematized Nomenclature of Medicine–Clinical Terms
SQL: Structured Query Language
