# Supplementary figures and images for: Bridging Data Models in Health Care With a Novel Intermediate Query Format for Feasibility Queries: Mixed Methods Study
Source: JMIR Med Inform. 2024 Oct 14;12:e58541. doi: 10.2196/58541 (PMC11493108; doi:10.2196/58541)

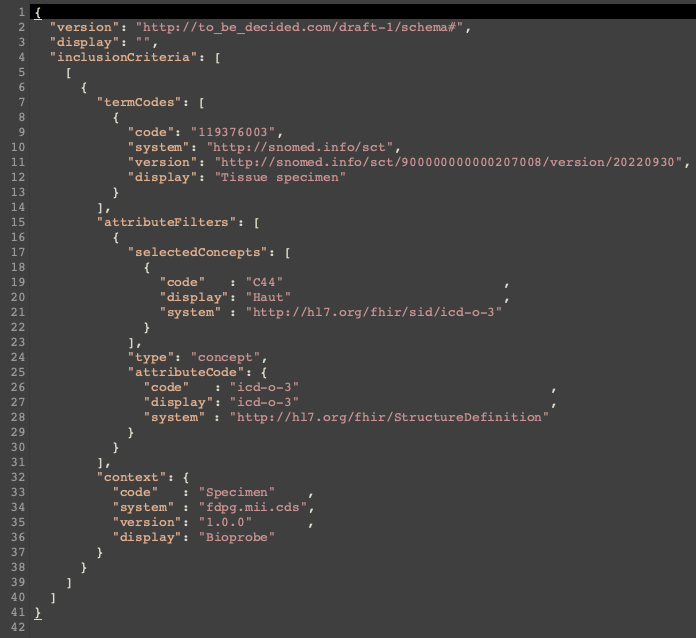

Supplement: Multimedia Appendix 1 [file medinform-v12-e58541-s001.png]
